# Elements of Hygiene and Public Health

**Published:** 1919

**Authors:** 


**Elements of Hygiene and Public Health.** By CHARLES PORTER, M.D., B.Sc., M.R.C.P. Edin. Pp. xiv., 411. London: Oxford Medical Publications. 1917. Price 12s. 6d. net.

A text-book for students and medical practitioners written by a physician who is also a barrister-at-law gives the ideal combination for a work on public health. It is not a compilation of laws, tables, statistics, or formulæ, but it is intended to enable the practitioner to answer the question of a healthy individual—How he is to be kept healthy? Attention is concentrated on causations, the individual causes, prevention—the concrete rather than the abstract—and ninety-eight excellent illustrations greatly increase the utility of the text. Twenty-three chapters on all the usual divisions of the whole subject of Sanitation give a concise description of the orthodox views of Sanitarians, and then follow short outlines on School Hygiene, Public Health Administration and Sanitary Laws. A few vital statistics and a very complete index conclude a well-written, well-printed, and well-illustrated volume, which cannot fail to be of much utility for consultation on the many questions of Sanitation which are commonly cropping up from day to day, and on which an authoritative statement is required.

We notice a remark appropriate to the present time outcry for eggs, "Though nutritious and digestible, they cannot be regarded as a cheap substitute for meat." It is obvious that at present prices they are the most expensive source of protein nourishment.

The book should be on the reading table of those whose motto is that of the sanitarian politician: "*Sanitas Sanitatum omnia Sanitas.*"

